# Hyperpolarization-Activated Current (I_h_) Is Reduced in Hippocampal Neurons from *Gabra5*−/− Mice

**DOI:** 10.1371/journal.pone.0058679

**Published:** 2013-03-14

**Authors:** Robert P. Bonin, Agnieszka A. Zurek, Jieying Yu, Douglas A. Bayliss, Beverley A. Orser

**Affiliations:** 1 Department of Physiology, University of Toronto, Toronto, Ontario, Canada; 2 Department of Pharmacology, University of Virginia Health System, Charlottesville, Virginia, United States of America; 3 Department of Anesthesia, University of Toronto, Toronto, Ontario, Canada; 4 Department of Anesthesia, Sunnybrook Health Sciences Centre, Toronto, Ontario, Canada; The Research Center of Neurobiology-Neurophysiology of Marseille, France

## Abstract

Changes in the expression of γ-aminobutyric acid type A (GABA_A_) receptors can either drive or mediate homeostatic alterations in neuronal excitability. A homeostatic relationship between α5 subunit-containing GABA_A_ (α5GABA_A_) receptors that generate a tonic inhibitory conductance, and HCN channels that generate a hyperpolarization-activated cation current (I_h_) was recently described for cortical neurons, where a reduction in I_h_ was accompanied by a reciprocal increase in the expression of α5GABA_A_ receptors resulting in the preservation of dendritosomatic synaptic function. Here, we report that in mice that lack the α5 subunit gene (*Gabra5*−/−), cultured embryonic hippocampal pyramidal neurons and ex vivo CA1 hippocampal neurons unexpectedly exhibited a decrease in I_h_ current density (by 40% and 28%, respectively), compared with neurons from wild-type (WT) mice. The resting membrane potential and membrane hyperpolarization induced by blockade of I_h_ with ZD-7288 were similar in cultured WT and *Gabra5*−/− neurons. In contrast, membrane hyperpolarization measured after a train of action potentials was lower in *Gabra5*−/− neurons than in WT neurons. Also, membrane impedance measured in response to low frequency stimulation was greater in cultured *Gabra5*−/− neurons. Finally, the expression of HCN1 protein that generates I_h_ was reduced by 41% in the hippocampus of *Gabra5*−/− mice. These data indicate that loss of a tonic GABAergic inhibitory conductance was followed by a compensatory reduction in I_h_. The results further suggest that the maintenance of resting membrane potential is preferentially maintained in mature and immature hippocampal neurons through the homeostatic co-regulation of structurally and biophysically distinct cation and anion channels.

## Introduction

Proper functioning of the central nervous system depends on the delicate control of neuronal excitability through a balance of excitation and inhibition. The homeostatic regulation of ion channels that regulate membrane conductance contributes to the maintenance of this balance [1], [2]. Pathological brain states can result when this balance is disrupted, such as the development of seizures following the loss of neuronal inhibition [3], [4]. Ample evidence suggests that homeostatic mechanisms exist to compensate for the loss of neuronal inhibition to maintain normal brain function [5], [6].

The neurotransmitter γ-aminobutyric acid (GABA) largely mediates inhibitory neurotransmission in the mammalian brain [7]. Activation of synaptically-localized type A GABA (GABA_A_) receptors results in rapid transient inhibition of postsynaptic neurons whereas activation of extrasynaptic GABA_A_ receptors by low concentrations of ambient GABA generates a tonic inhibitory conductance [8]. A tonic GABAergic conductance in the hippocampus is predominantly generated by GABA_A_ receptors that contain either the α5 subunit (α5GABA_A_) or δ subunit (δGABA_A_) [9], [10]. Tonic GABAergic inhibition can exert powerful regulatory constraints on neuronal firing, excitability, and plasticity of excitatory synapses of hippocampal pyramidal neurons [11]–[13].

Loss of tonic inhibition can induce compensatory changes in the expression of other ion channels that maintain normal neuronal function. For example, in cerebellar granule cells of α6GABA_A_ receptor-null mutant mice, the loss of tonic inhibition mediated by putative extrasynaptic δGABA_A_ receptors was accompanied by a homeostatic increase in the expression of two-pore domain K^+^ TASK-1 channels that generate a tonic inhibitory K^+^ current [14]. This increase in TASK-1 channel expression maintained neuronal excitability at levels observed in wild-type (WT) neurons.

Genetic deletion of voltage-dependent ion channels can also induce homeostatic changes in tonic GABAergic inhibition [15]. In particular, the genetic deletion of the hyperpolarization-activated cyclic nucleotide-gated type 1 (HCN1) channel which generates a hyperpolarization-activated cation current (I_h_) increased the expression of α5GABA_A_ receptors in cortical pyramidal neurons [15]. HCN channels are encoded by four genes (*HCN1–HCN4*), and are activated at hyperpolarized membrane potentials. HCN channels are permeable to both Na^+^ and K^+^ ions and mediate an inward current [16]. These non-inactivating ion channels exert complex effects on neuronal function by providing a tonic depolarizing current which contributes to resting membrane potential and opposes deviations away from the prevailing membrane potential. In hippocampal and neocortical pyramidal neurons, these biophysical properties of I_h_, together with a preferential distribution of the channels in distal dendrites limits the influence of excitatory synaptic input on membrane potential [17].

Pyramidal neurons of the hippocampus and cortex predominantly express the type-1 isoform of HCN (HCN1), and deletion of *HCN1* strongly decreases I_h_ in these neurons [18], [19]. Surprisingly, the summation of evoked excitatory post-synaptic potentials (EPSPs) in cortical neurons was unchanged following genetic deletion of HCN1 [15]. A homeostatic upregulation of α5GABA_A_ receptors in the cortex maintained the sublinear somatic summation of EPSPs following deletion of HCN1 [15]. As such, the increase in tonic inhibition compensated for the loss of I_h_ and constrained dendritosomatic efficacy. Notably, there was no upregulation of α5GABA_A_ receptors in hippocampal pyramidal neurons of *HCN1*−/− mice, perhaps due to a saturation of α5GABA_A_ receptor expression in these neurons [15].

α5GABA_A_ receptors and HCN1 channels have several common biophysical and functional properties that suggest they may mutually co-regulate neuronal excitability. For example, both channels can remain persistently activated following a hyperpolarization of the membrane to regulate resting membrane potential and conductance [11], [16], [20]. Additionally, HCN1 channels are expressed in high levels in the distal dendrites of hippocampal pyramidal neurons [21] where α5GABA_A_ receptors are also clustered [22]. Tonic inhibition and I_h_ both regulate the induction of long-term synaptic plasticity of hippocampal pyramidal neurons and limit sublinear EPSP summation in neocortical pyramidal neurons [15]. Finally, both α5GABA_A_ receptors and HCN1 channels constrain hippocampus-dependent memory performance [13], [19].

The functional commonalities between α5GABA_A_ receptors and HCN1 channels suggest that the potential reciprocal homeostatic co-regulation of these proteins is plausible. However, it is unknown whether the expression of α5GABA_A_ receptors regulates I_h_. In this study, we tested the hypothesis that a reduction in the expression of α5GABA_A_ receptors causes a reciprocal upregulation of I_h_ in hippocampal pyramidal neurons. Unexpectedly, we found the opposite, where a reduction in the expression of α5GABA_A_ receptors was associated with a reduction of I_h_ that contributes to homeostatic maintenance of resting membrane potential in these cells.

## Methods

### Electrophysiology

#### Hippocampal cell culture

The experiments reported here were approved by the Animal Care Committee of the University of Toronto. All experiments were conducted with hippocampal tissue harvested from WT *Gabra5+/+* or α5GABA_A_ null mutant mice (*Gabra5*−/−) mice. Generation of the *Gabra5*−/− mice has been described previously [23]. Briefly, all mice were of mixed genetic background (50:50 C57BL/6 and 129SvEv), and WT and *Gabra5*−/− mice were generated by crossing heterozygous *Gabra5*+/− mice. Cultures of hippocampal neurons were prepared as previously described [11] from *Gabra5*−/− and WT littermates on postnatal day 1. Cells were maintained in culture for 14 to 21 days before experimentation.

#### Hippocampal brain slices

Slices were prepared from WT and *Gabra5*−/− mice that ranged in age from postnatal day 17–21. After administration of isoflurane anesthesia, the mice were decapitated and their brains quickly removed and placed in ice-cold, oxygenated (95% O_2_, 5% CO_2_) artificial cerebrospinal fluid (aCSF; containing in mM: NaCl 124, KCl 3, MgCl_2_ 1.3, CaCl_2_ 2.6, NaH_2_PO_4_ 1.25, NaHCO_3_ 26, d-glucose 10) with osmolarity adjusted to 300–310 mOsm. Brain slices (350 µm) containing coronal sections of the hippocampus were prepared with a VT1200 tissue slicer (Leica, IL, USA).

#### Data Acquisition

Data were acquired with a Multiclamp 700B amplifier (Molecular Devices Corporation, Sunnyvale, CA, USA) controlled with pClamp 9.0 software (Molecular Devices Corporation) via a Digidata 1322 interface (Molecular Devices Corporation). Membrane current and voltage were filtered at 2 kHz and sampled at 10 kHz for all electrophysiological experiments. Membrane capacitance was measured with the membrane test protocol in pClamp 9.0. Access resistance was monitored periodically throughout the experiments by a brief 10-mV or 10-pA hyperpolarizing step during voltage-clamp and current-clamp experiments, respectively. Cells were eliminated from further analysis if the access resistance changed by more than 20% over the recording period. Liquid junction potential and pipette capacitance were corrected using the pClamp 9.0 software before the whole-cell configuration was established.

Patch pipettes, pulled from thin-walled borosilicate glass capillary tubes, had open-tip resistances of 4 to 6 MΩ when filled with an intracellular solution that contained (in mM) 145 K^+^ gluconate, 5 Na^+^ gluconate, 2 KCl, 10 HEPES, 11 EGTA, 4 Mg^2+^ATP, and 1 CaCl_2_ with an osmolarity of 300 to 320 mOsm and the pH adjusted to 7.3 with KOH. Extracellular solutions for all experiments contained (in mM) 140 NaCl, 1.3 CaCl_2_, 2.0 KCl, 25 HEPES, and 33 glucose; the osmolarity was adjusted to 290 to 300 mOsm with sucrose, and the pH was adjusted to 7.4 with 10 N NaOH. The extracellular solution was applied directly to neurons at a rate of 1 ml/min by a computer-controlled, multi-barreled perfusion system (SF-77B; Warner Instruments, Hamden, CT, USA). Whole-cell current was recorded with the holding potential clamped at −60 mV except where indicated otherwise.

Experiments in cultured pyramidal neurons were performed as previously described [11]. For experiments in hippocampal slices, whole-cell recordings were obtained from the pyramidal cell layer using a blind-patch technique. Neurons with small membrane capacitances suggestive of non-pyramidal neurons in this preparation (<60 pF) were excluded from study (3 WT, 1 *Gabra5* −/− neuron). The composition of the intracellular solution and the recording procedures were identical to those described for the recordings from cultured neurons.

In all experiments, the ionotropic glutamate antagonists 6-cyano-7-nitroquinoxaline-2,3-dione (10 µM) and 2-amino-4-phosphonovaleric acid (40 µM) were added to the extracellular solution. In experiments designed to measure I_h_ and membrane impedance, the Na^+^ channel blocker tetrodotoxin (0.3 µM; Alomone Labs, Jerusalem, Israel) was added to the extracellular solution. Aqueous stock solutions of all drugs were prepared with distilled water. All drugs and chemicals were purchased from Sigma-Aldrich (Oakville, Ontario, Canada) except where indicated otherwise.

#### Measurement of I_h_

**Figure 1:**
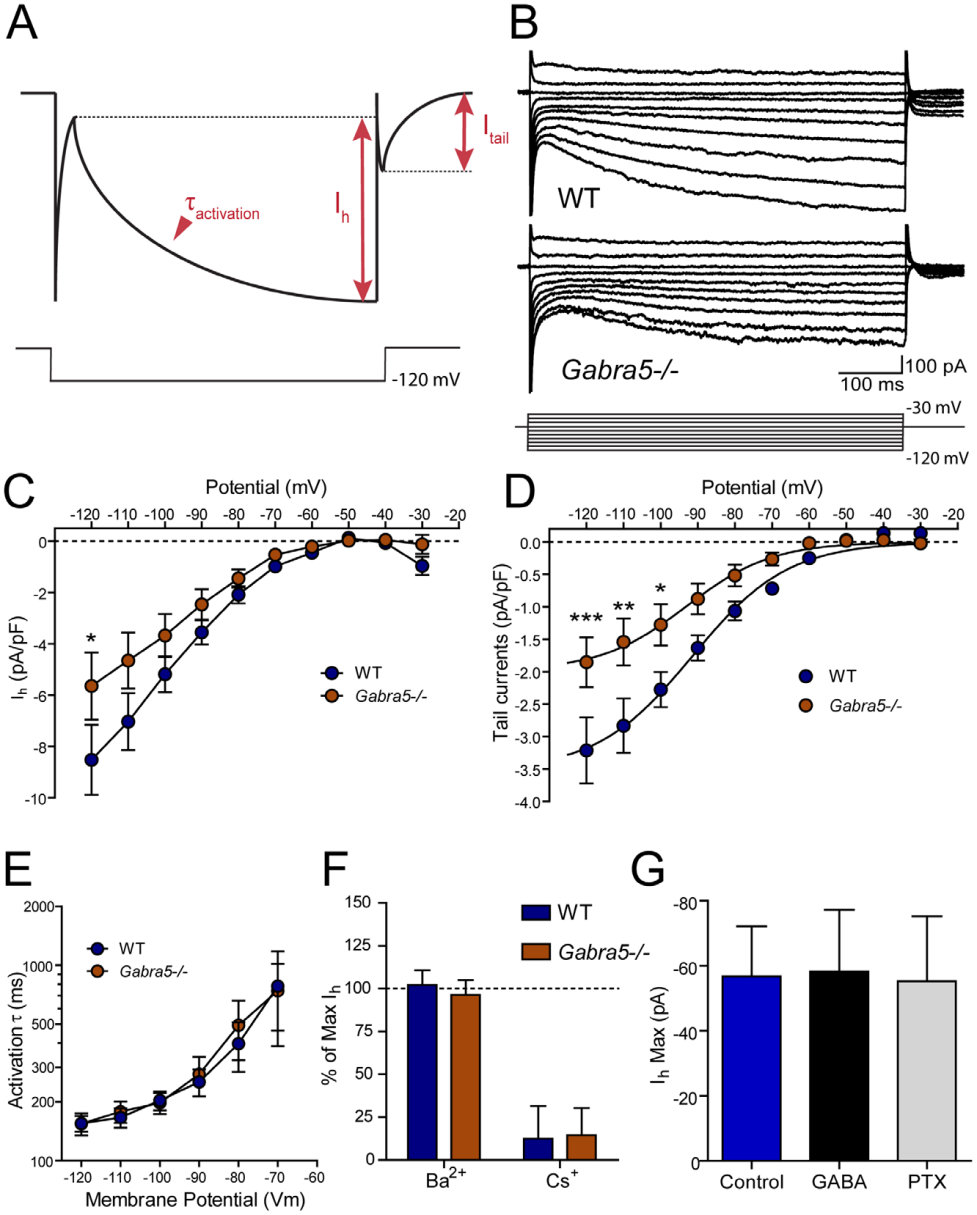
Reduced I_h_ in cultured *Gabra5*−/− neurons. A) Schematic illustrating the method of I_h_ measurement B) I_h_ was activated in cultured hippocampal pyramidal neurons of wild-type (WT) and *Gabra5*−/− neurons by changing the membrane potential from −120 mV to −30 mV in 10-mV increments. C) Estimation of I_h_ conductance from the linear portion of the current-voltage curve generated by hyperpolarizing the resting membrane potential revealed a 43% reduction of I_h_ conductance in *Gabra5*−/− neurons. D) Quantification of the I_h_ tail currents that remained after membrane potential was returned to −60 mV revealed significantly lower I_h_ density in *Gabra5*−/− neurons (n = 16) than in WT neurons (n = 9). Neither the kinetics of I_h_ activation (E) nor sensitivity to Ba^2+^ (0.5 mM; n = 5) or Cs^+^ (0.5 mM; n = 4) (F) were changed in *Gabra5*−/− neurons, which suggested no change in the subtypes of HCN channels generating I_h_. G) Enhancing or reducing the tonic current in WT neurons with 1 µM GABA (n = 6) or 1 µM picrotoxin (PTX; n = 6), respectively, did not change I_h_ measured at −120 mV, demonstrating that the lower level of I_h_ in *Gabra5*−/− neurons is independent of changes in tonic inhibition.

I_h_ was activated by changing the holding potential from −60 mV through a range of test potentials (from −120 mV to −30 mV) in 10-mV steps. Each test potential was maintained for 500 ms. The net I_h_ conductance was measured as the difference between the steady-state current at the end of the test potential and the minimum current measured within 100 ms of the start of the test potential (Fig 1A). The I_h_ tail current was measured as the peak amplitude of the residual current measured at the end of each test potential immediately after the return the holding potential to −60 mV. The membrane potential that evoked half-maximal activation (V_50_) of I_h_ was determined by fitting the tail current activation data to a Boltzmann sigmoidal function using Graphpad 4 (Graphpad, San Diego, CA, USA). The kinetics of I_h_ activation, measured at holding potentials between −120 mV and −70 mV, were determined by fitting onset of the current with a single exponential curve using Clampfit 10 (Molecular Devices Corporation) with the equation: 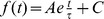. The net I_h_ was measured at the end of the test holding potential, and the I_h_ conductance was estimated by fitting the net I_h_ measured between −120 mV and −90 mV with a linear regression line.

#### Measurement of after-hyperpolarization

An after-hyperpolarization of the membrane was induced by stimulating neurons with a train of action potentials in current-clamp mode. A depolarizing current sufficient to stimulate action potential firing at a frequency of 5 Hz for 2 s was applied and after-hyperpolarization was measured as the area under the curve, relative to resting membrane potential, of the membrane potential over the period of hyperpolarization following the train of action potentials. The decay time constant (τ) of the after-hyperpolarization was measured with Clampfit 10 by fitting the decay with a standard single exponential curve.

#### Determination of membrane impedance

The neuronal frequency-dependent membrane impedance was studied using the impedance (Z) amplitude profile (ZAP) as described previously [24]. In brief, in whole-cell current-clamp mode, neurons were injected with a sinusoidal current of constant amplitude and linearly increasing frequency (0–40 Hz over 30 s). The amplitude of the ZAP current was adjusted to maintain a peak depolarization of the membrane potential of approximately 10 mV positive to resting potential. The frequency-dependent membrane impedance was determined by transforming the membrane voltage and input current recordings with a fast Fourier transform over the range of frequencies from 0.5 to 40 Hz with Clampfit 10 and dividing the transformed voltage by the current. The peak resonance frequency was determined as the input frequency at which membrane resistance was the greatest.

### Western blot measurements

Hippocampal tissue was collected from adult (16 weeks old) WT and *Gabra5*−/− mice. Hippocampal tissue was dissected from whole brains in ice cold phosphate-buffered saline (pH 7.4) and homogenized with a Dounce homogenizer (Wheaton, NJ, USA). The homogenization buffer contained (in mM): 10 Tris-HCl, 5 NaF, 1 Na_3_VO_4_, 1 EDTA, 1 EGTA, 320 sucrose, protease inhibitor EDTA free tablet (Roche Diagnostics, Germany) at pH 7.4. The homogenate was centrifuged at 900 g for 10 min at 4°C, and the supernatant was spun again at 10,000 g for 20 min. The final supernatant was isolated in lysis buffer containing (in mM): 20 Tris-HCl, 150 NaCl, 5 EDTA, 10 NaF, 2 Na_3_VO_4_, 10 sodium pyrophosphate, 1% (v/v) Triton X-100, and 0.1% (w/v) sodium dodecyl sulfate (SDS), EDTA-free protease inhibitor tablet (Roche Diagnostics, Germany). The supernatant was homogenized using a probe sonicator (Cole Parmer Instruments, IL, USA) and protein concentration was determined using bicinchoninic acid assay (BCA) (Thermo Scientific, IL, USA).

Hippocampal protein (15 µg) was loaded on 10% Bis-Tris gels and transferred onto nitrocellulose membranes (Pall Life Sciences, NY, USA) followed by SDS-PAGE. The membranes were rinsed in TBS-Tween that contained 50 mM Tris-HCl, 150 mM NaCl, and 0.05% (v/v) Tween 20 and then incubated in 5% (w/v) milk in TBS-Tween at room temperature for 1 hr. Primary and secondary antibodies were diluted in 3% (w/v) bovine serum albumin in TBS-Tween. The membranes were incubated with 1∶1000 anti-HCN1 antibody (clone N70/28; NeuroMab, UC Davis NeuroMab facility, CA, USA) overnight at 4°C, washed with TBS-Tween, and incubated in 1∶1000 anti-mouse antibody (Cell Signaling, MA, USA) at room temperature for 1 hr. The membranes were treated with enhanced chemiluminesence western blotting substrate (Thermo Scientific, IL, USA) for protein band visualization. HCN1 primary and secondary antibodies were stripped from the membranes by incubating in stripping buffer (Thermo Scientific, IL, USA) at room temperature for 20 min, followed by 4 washes in TBS-Tween. To allow the normalization of HCN1 blot densities, β-actin blots were then performed using the western blotting procedure described above with 1∶1000 anti-β-actin antibody (Millipore, MA, USA), followed by 1∶1000 anti-rabbit antibody (Cell Signaling, MA, USA).

All membranes were exposed and quantified using the Kodak Image Station 2000R (Kodak, USA). Because HCN1 is known to exist in a glycosylated (108 kDa) and unglycosylated (100 kDa) form, both of which are recognized by the Anti-HCN1 antibody used (clone N70/28; NeuroMab), the densities of both bands were pooled for analysis as described elsewhere [25]. The density of HCN1 bands were normalized to β-actin, a prototypical loading control.

### Statistical analyses

Statistical analyses were performed using Graphpad Prism 4. Membrane impedance and I_h_ tail current and activation kinetics were analyzed with two-way repeated-measures ANOVA followed by a Bonferroni post hoc test. The remaining comparisons were performed with one-way ANOVA or Student t-tests, as appropriate. Any p value less than 0.05 was considered significant. All data are shown as mean ± standard error of the mean.

## Results

The biophysical properties of cultured hippocampal neurons from WT and *Gabra5*−/− mice were studied using standard whole-cell patch clamp techniques. WT and *Gabra5*−/− neurons had similar membrane capacitances (WT: 33.5 pF±2.0 pF, n = 16; *Gabra5*−/−: 35.0 pF±1.6 pF, n = 14; p>0.05) and resting membrane potentials (WT: −67.5 mV±0.8 mV, n = 16; *Gabra5*−/−: −67.9 mV±0.7 mV, n = 14; p>0.05), as shown previously [11]. However, *Gabra5*−/− neurons had higher input resistances than WT neurons (WT: 231 MΩ±9 MΩ, n = 16; *Gabra5*−/−: 313 MΩ±11 MΩ, n = 14; p<0.0001), owing in part to the loss of tonic inhibition as described in a previous report [11].

### Reduced I_h_ in cultured *Gabra5*−/− neurons

Next, the amplitude of the I_h_ current was measured in WT and *Gabra5*−/− neurons (Fig 1A) The net I_h_ was measured as the time-dependent inward current activated by the voltage step (Fig 1B). The I_h_ conductance was estimated from the near linear current-voltage relationship of I_h_ measured between −120 mV and −90 mV (Fig 1C). From these analyses, the total I_h_ conductance was estimated to be 43% smaller in *Gabra5*−/− neurons compared with WT neurons (WT: 6.0 nS±0.2 nS, n = 9; *Gabra5*−/−: 3.4 nS±0.1 nS, n = 16; p<0.0001). The maximum amplitude of the tail current measured following the hyperpolarizing voltage steps was smaller in *Gabra5*−/− neurons (n = 16) than in WT neurons (n = 9; Fig 1D; voltage × genotype: F_9,198_ = 4.09; p<0.0001), consistent with a reduced I_h_ in these neurons. The HCN channel blocker ZD-7288 caused a complete block of I_h_ in both WT and *Gabra5*−/− neurons (data not shown).

The reduced I_h_ in *Gabra5*−/− neurons may result from the substitution of HCN1 with another HCN isoform. The subtype of HCN channels determines its sensitivity to cAMP and voltage-dependent activation and kinetics [16]. Thus, a substitution of HCN subtype is predicted to be accompanied by changes in the activation kinetics and voltage-dependent activation of I_h_. However, we observed that the time course of current activation (τ I_h_) was similar between WT and *Gabra5*−/− neurons (Fig 1E) (genotype × voltage: F_5,99_ = 0.05, p>0.05). In addition, the voltage-sensitivity of I_h_, measured as the half-maximal activation voltage (V_50_) of the tail currents (Fig 1C), was similar between WT and *Gabra5*−/− mice (WT: −91.5 mV±5.0 mV, n = 9; *Gabra5*−/−: −93.3 mV±7.3 mV, n = 16, p>0.05). These results suggest that the lower I_h_ in *Gabra5*−/− neurons is not likely due to a change in the subpopulation of HCN channels that generate I_h_.

A pharmacological characteristic of I_h_ generated by HCN channels is an insensitivity to low concentrations of extracellular barium and potent inhibition induced by low concentrations of extracellular cesium [26]. To confirm that the reduction in I_h_ in *Gabra5*−/− neurons resulted from a decrease in HCN-generated current; we applied a low concentrations of either BaCl_2_ (0.5 mM) or CsCl (0.5 mM). Consistent with HCN pharmacology, BaCl_2_ (0.5 mM) did not block I_h_ in WT (n = 5) or *Gabra5*−/− (n = 5) neurons, but CsCl (0.5 mM) caused near complete inhibition of I_h_ in both WT (n = 4) and *Gabra5*−/−−/− (n = 4) neurons, when I_h_ was activated at −120 mV (Fig 1F).

We next sought to determine whether the acute enhancement or inhibition of α5GABA_A_ receptor-mediated current changed I_h_, similar to the reduction of I_h_ observed following genetic deletion of α5GABA_A_ receptors. The tonic current was either enhanced by applying 1 µM GABA (n = 6) or inhibited by applying 1 µM picrotoxin (n = 6), as described previously [11] then I_h_ was activated in WT neurons by hyperpolarizing the membrane potential to −120 mV. Neither enhancement or inhibition of the tonic current changed the amplitude of I_h_ (Fig 1G; one-way ANOVA F_2,18_ = 0.08, p>0.05).

I_h_ can exert a powerful regulatory effect on the resting membrane potential of neurons [16]. Further, the dynamic voltage-dependent activity of depolarizing I_h_ opposes any changes in membrane potential away from the resting membrane potential. We next sought to determine whether the lower I_h_ in *Gabra5*−/− neurons would exert less control over resting membrane potential than in WT neurons. Application of the HCN antagonist ZD-7288 (20 µM) induced a similar hyperpolarization of the resting membrane potential of approximately 5.5 mV in both WT and *Gabra5*−/− neurons (WT + ZD-7288: −72.8 mV±2.0 mV, n = 8; *Gabra5*−/− + ZD-7288: −73.6 mV±1.8 mV, n = 7, p>0.05). These results suggest that the baseline level of I_h_ depolarized the resting membrane potential to a similar degree in both WT and *Gabra5*−/− neurons.

### Reduced after-hyperpolarization in cultured *Gabra5*−/− neurons

**Figure 2:**
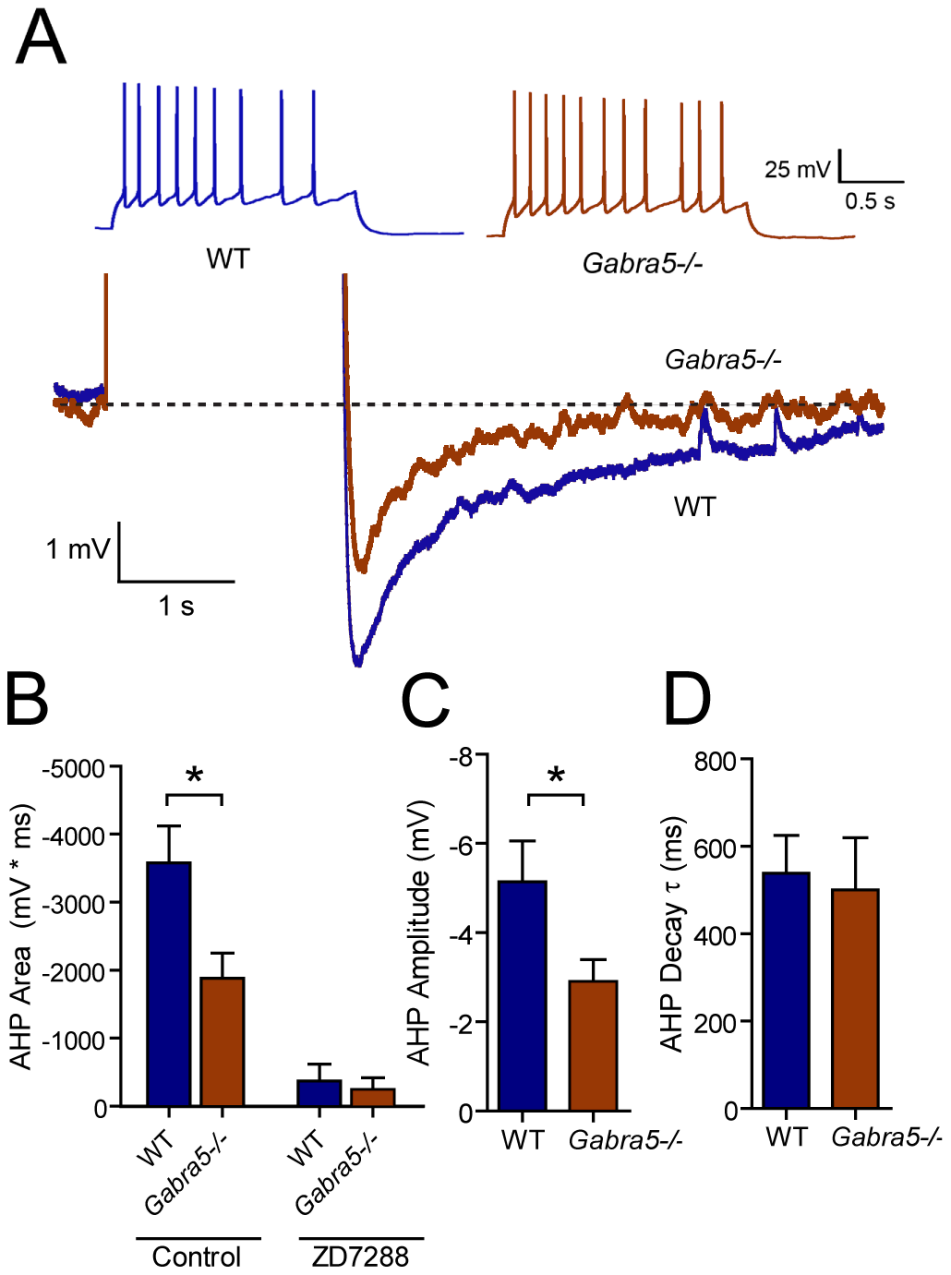
Reduced after-hyperpolarization in cultured *Gabra5*−/− neurons. A) Action potentials were elicited in WT and *Gabra5*−/− neurons at a frequency of 5 Hz (examples shown in upper traces). The membrane hyperpolarization that occurred following depolarization was measured relative to resting membrane potential to reveal a reduced after-hyperpolarization in *Gabra5*−/− neurons (example traces enlarged to emphasize after-hyperpolarization are shown in lower traces). B) The area of after-hyperpolarization was smaller in *Gabra5*−/− neurons (n = 9) than in WT neurons (n = 12). Application of the HCN antagonist ZD-7288 (20 µM) blocked the after-hyperpolarization in neurons of both genotypes (n = 5), confirming the contribution of I_h_ to after-hyperpolarization. C) The peak after-hyperpolarization, measured relative to the resting membrane potential, was also smaller in *Gabra5*−/− neurons compared to WT. D) The decay kinetics of after-hyperpolarization were similar between WT and *Gabra5*−/− neurons.

**Figure 3:**
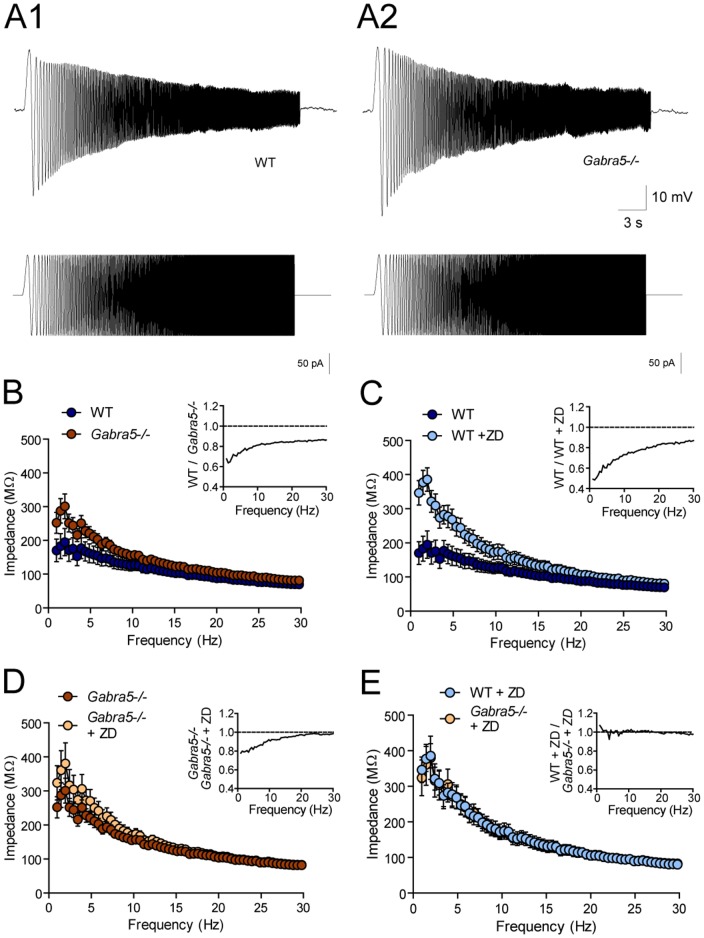
Increased membrane impedance in response to low-frequency input in cultured *Gabra5*−/− neurons. A) The membrane impedance properties of WT and *Gabra5*−/− neurons were determined by quantifying membrane resistance during the injection of a sinusoidal current ranging in frequency from 0 to 40 Hz. Example traces show larger changes in membrane potential in the *Gabra5*−/− neuron at low frequencies, indicative of an increased membrane impedance. B) The membrane impedance of *Gabra5*−/− neurons (n = 10) was greater than that of WT neurons (n = 7) in response to low-frequency input from 1 to 4 Hz (p_1.0 Hz_<0.01, p_1.5 Hz_<0.001, p_2.0 Hz_<0.001, p_2.4 Hz_<0.01, p_2.9 Hz_<0.05, p_3.4 Hz_>0.05, p_3.9 Hz_<0.01). The inset shows the membrane impedance ratio of *Gabra5*−/− to WT neurons. C) Blockade of I_h_ in WT neurons with ZD-7288 (n = 4) increases membrane impedance to input from 1 to 6 Hz (p_1.0 Hz_<0.001, p_1.5 Hz_<0.001, p_2.0 Hz<_0.001, p_2.4 Hz_<0.001, p_2.9 Hz_<0.001, p_3.4 Hz_<0.001, p_3.9 Hz<_0.001, p_4.4 Hz_<0.001, p_4.9 Hz_<0.001, p_5.4 Hz_<0.05, p_5.9 Hz_<0.05). D) Blockade of I_h_ in *Gabra5*−/− neurons with ZD-7288 (n = 5) caused a modest but significant increase in membrane impedance. Post hoc analysis did not reveal significant differences within any specific frequency range. E) No differences were observed in the impedance of *Gabra5*−/− and WT neurons in the presence of ZD-7288. Asterisks indicating significant differences within specific frequency ranges have been omitted for clarity.

Following a train of action potentials, the membrane potential is often hyperpolarized below the resting potential, in part due to deactivation of I_h_ [27]. This after-hyperpolarization is a key determinant of spike frequency adaptation in hippocampal neurons [28], [29]. We next studied the potential functional consequences of the reduced I_h_ conductance on neuronal after-hyperpolarization. Neurons were depolarized to fire action potentials at a rate of 5–6 Hz for 2 s, and the after-hyperpolarization was measured as the area of the subsequent membrane hyperpolarization (i.e., after the action potential train) (Fig 2A). The amount of excitatory current required to produce similar frequencies of action potentials was lower in *Gabra5*−/− neurons than in WT neurons (WT: 7.67 pA/pF±1.00 pA/pF, n = 12; *Gabra5*−/−: 4.81 pA/pF±0.64 pA/pF, n = 9; p = 0.038) as reported previously [11]. The resting membrane potential was similar between WT and *Gabra5*−/− neurons (WT: −68.0 mV±1.4 mV, n = 12; *Gabra5*−/−: −68.8 mV±1.2 mV, n =  9; p>0.05). Application of ZD-7288 (20 µM) abolished the after-hyperpolarization in both WT and *Gabra5*−/− neurons (Fig 2B), which confirmed the important role of I_h_ in the after-hyperpolarization. The after-hyperpolarization was smaller in *Gabra5*−/− than in WT neurons (Fig 3B) (WT: −3528 mV·s±540 mV·s, n = 12; *Gabra5*−/−: −1883 mV·s±369 mV·s, n = 9; p = 0.022). This effect was largely due to a reduction in peak after-hyperpolarization potential seen in *Gabra5*−/− neurons compared to WT (Fig 2C) (WT: −5.1 mV±0.8 mV, n = 12; *Gabra5*−/−: −2.9 mV±0.5 mV, n = 9; p = 0.040). However, the τ of the after-hyperpolarization did not differ between WT and *Gabra5*−/− neurons (Fig 2D) (WT: 538 ms±86 ms, n = 12; *Gabra5*−/−: 501 ms±119 ms, n = 9; p>0.05), consistent with similar I_h_ kinetics in these neurons.

### Increased low-frequency membrane impedance in *Gabra5*−/− neurons

**Figure 4:**
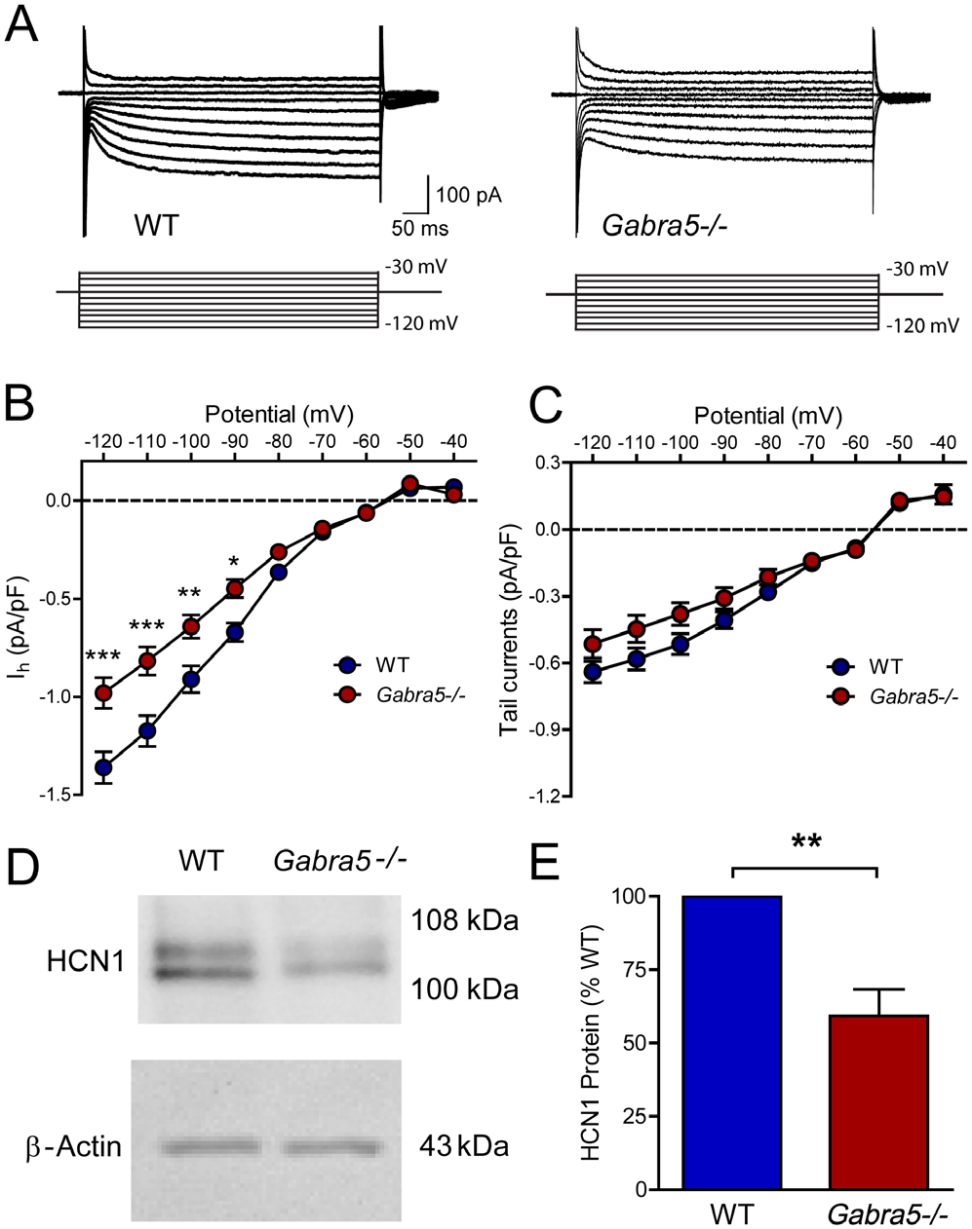
Reduced I_h_ and HCN1 expression in hippocampus of *Gabra5*−/− mice. A) Representative traces of I_h_ in CA1 pyramidal neurons in hippocampal slices obtained from postnatal WT and *Gabra5*−/− mice. I_h_ was activated and measured by changing the membrane potential from −120 mV to −30 mV in 10-mV increments. B) Estimation of I_h_ conductance from the linear portion of the current-voltage curve revealed a 28% reduction of I_h_ in *Gabra5*−/− neurons. C) A modest but significant reduction in I_h_ tail current was also observed in *Gabra5*−/− neurons. Post hoc analysis did not reveal significant differences at any specific test potential. D) The expression of HCN1 protein and β-actin in hippocampal tissue from adult WT and *Gabra5*−/− mice. E) After normalization to β-actin, the expression of HCN1 was reduced in hippocampal tissue from *Gabra5*−/− mice by 41% relative to WT mice, paralleling the decrease in I_h_ current.

Previous studies have demonstrated that I_h_ contributes to the frequency-dependent membrane impedance of neurons [19], [24]. Specifically, I_h_ generated by HCN1 in hippocampal pyramidal neurons selectively attenuates changes in membrane potential resulting from low-frequency input (< 5Hz), which in turn reduces the subthreshold membrane resonance properties of neurons in response to input in this frequency range [19], [24]. We examined the membrane impedance properties of cultured WT (n = 7) and *Gabra5*−/− (n = 10) neurons by injecting an oscillating current of linearly increasing frequency and then measuring the impedance (Fig 3A). *Gabra5*−/− neurons had a higher frequency-dependent impedance than WT neurons in response to stimulation in the frequency range of 0 to 4 Hz (Fig 3B) (genotype × frequency: F_80,1215_ = 1.39; p = 0.016). Post hoc analysis revealed a significantly greater membrane impedance in *Gabra5*−/− than in WT neurons over most of the frequency range from 1 to 4 Hz (Fig 4B).

To ascertain whether this difference in membrane impedance between WT and *Gabra5*−/− resulted from the lower I_h_, we tested for changes in membrane impedance following the application of ZD-7288 (20 µM). In WT neurons (n = 4), blockade of I_h_ by ZD-7288 resulted in a robust frequency-dependent increase in membrane impedance (Fig 3C) (genotype × frequency: F_80,729_ = 3.35; p<0.0001). Post hoc analysis of this interaction revealed a significant increase in membrane impedance in the frequency range 1 to 6 Hz (Fig 3C). In contrast, ZD-7288 caused only a modest frequency-dependent increase in membrane impedance in *Gabra5*−/− neurons (n = 5), which was not significantly different from control at any specific frequency (Fig 3D) (main effect of drug: F_1,1053_ = 32.05, p<0.0001). Notably, the membrane impedance of WT and *Gabra5*−/− neurons was similar following application of ZD-7288 (Fig 3E) (main effect of genotype: F_1,567_ = 0.006; p>0.05). Finally, the peak resonant frequency of the neurons was similar in both genotypes and drug conditions (WT: 1.15 Hz±0.03 Hz; *Gabra5*−/−: 1.20 Hz±0.03 Hz; WT + ZD-7288: 1.13 Hz±0.03 Hz; *Gabra5*−/− + ZD-7288: 1.18±0.06. Two way ANOVA genotype: F_1,22_ = 1.67, p<0.05; drug: F_1,22_ = 0.268, p>0.05). The similarities between the membrane impedance of WT and *Gabra5*−/− neurons in the presence of ZD-7288 suggest that the tonic GABAergic inhibitory conductance does not significantly contribute to membrane impedance properties of pyramidal neurons.

### Reduced I_h_ and HCN1 in *Gabra5*−/− hippocampal neurons in brain slices

We next sought to determine whether the reduced I_h_ observed in cultured *Gabra5*−/− hippocampal neurons was also present in neurons of the hippocampal CA1 pyramidal layer recorded in brain slices (Fig 4A). Similar to cultured neurons, we observed an increased membrane resistance in *Gabra5*−/− neurons compared to WT (*Gabra5*−/−: 212 MΩ±14 MΩ, n = 13; WT: 158 MΩ±19 MΩ, n = 12; p = 0.027). I_h_ current density was again reduced in *Gabra5*−/− neurons (n = 12) compared to WT (n = 8) (Fig 4B) (voltage × genotype: F_8,144_ = 9.64; p<0.0001). Relative to WT neurons, the total I_h_ conductance was estimated to be 28% lower in *Gabra5*−/− neurons (WT: 4.5 nS±0.3 nS, n = 8; *Gabra5*−/−: 3.2 nS±0.4 nS, n = 12; p = 0.030). I_h_ tail current was also reduced in *Gabra5*−/− neurons compared to WT (voltage × genotype: F_8,144_ = 3.03; p = 0.004), although post-hoc analysis did not reveal a significant reduction at any specific potential (Fig 4C). The difference in I_h_ current density was not attributable to differences in cell size (WT: 166 pF±23 pF; *Gabra5*−/−: 196 pF±14 pF; p = 0.30). Additionally, the V_50_ of I_h_ was similar between WT and *Gabra5*−/− mice (WT: −84.2 mV±3.4 mV, n = 8; *Gabra5*−/−: −84.6 mV±3.0 mV, n = 12; p>0.05). These data suggest that the reduction of I_h_ in *Gabra5*−/− neurons is robust and occurs at different stages of development and in different neuronal environments.

### Protein levels of HCN1 are reduced in *Gabra5*−/− neurons

One likely explanation for the reduction of I_h_ in *Gabra5*−/− neurons, in the absence of changes in I_h_ kinetics, is a decrease in the expression of HCN1 protein. This hypothesis was tested by measuring levels of HCN1 protein in hippocampal tissue samples from adult WT (n = 6) and *Gabra5*−/− (n = 6) mice (Fig 4D). HCN1 was selected for measurement since it is the most highly expressed isoform in the hippocampus CA1 [21]. Densitometric analysis showed that compared to WT mice, total protein expression of HCN1 in the hippocampus of *Gabra5*−/− mice was decreased by 40.8%±9.1% (Fig 4E) (one-sample t-test, p = 0.002). Thus, the magnitude of the reduction of HCN1 protein in *Gabra5*−/− hippocampal neurons closely paralleled the reduction of I_h_.

## Discussion

Here, we tested the hypothesis that reduced expression of α5GABA_A_ receptors would be accompanied by a reciprocal increase in I_h_ [15]. Unexpectedly, we observed a reduction in I_h_ in *Gabra5*−/− hippocampal neurons compared to WT neurons, as indicated by the lower hyperpolarization-activated current, lower after-hyperpolarization, and greater low-frequency membrane impedance. The reduction in I_h_ was observed in both cultured neurons and in hippocampal pyramidal neurons. We observed no change in I_h_ activation kinetics in *Gabra5*−/− neurons, suggesting that changes in HCN channel isoform did not contribute to the reduced I_h_ in *Gabra5*−/− neurons. Finally, we observed a decrease in the protein levels of HCN1 in *Gabra5*−/− hippocampus that paralleled the reduction of I_h_ observed in *Gabra5*−/− neurons.

### Reduced I_h_ maintains normal resting membrane potential in *Gabra5*−/− neurons

The resting membrane potential was not different in *Gabra5*−/− neurons, despite the fact that the tonic inhibitory conductance generated by α5GABA_A_ receptors was absent in *Gabra5*−/− neurons [11]. These data raise the possibility that a decrease in I_h_, which normally provides a tonic depolarizing current, serves to homeostatically maintain the same resting membrane potential in *Gabra5*−/− and WT neurons. It is notable that the reduced I_h_ current associated with deletion of the α5GABA_A_ receptor was observed in both cultured hippocampal pyramidal neurons and in CA1 hippocampal neurons. This finding suggests that there exists a robust relationship between α5GABA_A_ receptor and HCN1 channel expression that persists in very different neuronal environments and at different developmental stages.

The lack of change in resting membrane potential contrasted with the differences between WT and *Gabra5*−/− mice in after-hyperpolarization and membrane impedance. The after-hyperpolarization was reduced in *Gabra5*−/− neurons. Since ZD-7288 blocked the after-hyperpolarization in both WT and *Gabra5*−/− neurons, the after-hyperpolarization measured here was predominantly generated through the voltage-dependent deactivation of I_h_ during depolarization. Despite the differences in peak after-hyperpolarization, activation of I_h_ terminated the after-hyperpolarization similarly in WT and *Gabra5*−/− neurons. Because of the role I_h_ plays in regulating the firing of action potentials [28], a reduced after-hyperpolarization may disturb the firing frequency of *Gabra5*−/− neurons. Nonetheless, the reduced I_h_ in *Gabra5*−/− neurons appears to maintain membrane potential even at the expense of a reduced after-hyperpolarization and the potential consequences on firing activity.

A reduction in I_h_ also increased the frequency-dependent membrane impedance in *Gabra5*−/− neurons. These findings are consistent with the established role of I_h_ in reducing membrane impedance to low-frequency, fluctuating input [19], [24]. Similar to after-hyperpolarization, we found that membrane impedance was not greatly influenced by tonic α5GABA_A_ receptor activity, since WT and *Gabra5*−/− neurons exhibit similar membrane impedances when I_h_ was blocked by ZD-7288. Overall our data suggest that the reduced I_h_ in *Gabra5*−/− hippocampal neurons homeostatically maintains resting membrane potential, with consequential changes in other neuronal properties and behaviours that are regulated by I_h_, such as after-hyperpolarization and membrane impedance. Whether the reduced I_h_ also restores normal synaptic integration in *Gabra5*−/− neurons [15] remains to be determined.

### Homeostasis of neuronal excitability following reduction of tonic GABAergic inhibition

Deletion of the GABA_A_ receptors that contribute to tonic GABAergic inhibition causes changes in other conductances that regulate neuronal excitability. For example, the genetic deletion of α6GABA_A_ receptors, which mediate a tonic current in cerebellar granule cells, causes the upregulation of the two-pore-domain leak K^+^ channel, TASK-1 [14]. The converse relationship has also been found: genetic deletion of Kv4.2 K^+^ channels was associated with an increased tonic inhibitory current in hippocampal pyramidal neurons [30]. In both of these examples, the loss of one inhibitory current was offset by an increase in another inhibitory current to maintain normal neuronal excitability. We showed that the genetic deletion of α5GABA_A_ receptors that generate tonic outward currents in hippocampal neurons [9] was associated with a decrease in I_h_ that provides tonic inward current. As such, the normal relative levels of outward and inward current could be maintained, as reflected in the lack of difference in resting membrane potential between WT and *Gabra5*−/− neurons.

It is notable that in previous studies, an upregulation of α5GABA_A_ receptors was not observed in hippocampal pyramidal neurons of *HCN1*−/− mice [15]. The expression of α5GABA_A_ receptors in the hippocampus is among the highest in the mammalian brain [31]. The high basal level of expression of α5GABA_A_ receptors may reduce or eliminate the capacity for further upregulation of these receptors [15]. Alternatively, HCN1 channels and α5GABA_A_ receptors may serve different functional roles in hippocampal pyramidal neurons and may be homeostatically co-regulated in a manner different from that observed in cortical neurons. The cortex and hippocampus are distinct neuronal environments that may exert unique homeostatic pressures, such that either resting membrane potential or EPSP summation is preferentially preserved through compensatory mechanisms [15]. Thus, the mechanisms of compensation may be diverse and likely vary depending on the primary contribution of the ionic currents to neuronal function and the prevailing activity patterns of the neurons [32], [33]. Finally, tonic inhibitory currents are subject to regulation by endogenous hormones, such as neuroactive steroids and insulin [34], [35]. It would be of interest to ascertain whether the endogenous regulation of tonic inhibition also induces changes in I_h_.

Lastly, HCN1 channels expressed in hippocampal CA1 pyramidal neurons play an important role in the regulation of hippocampus-dependent memory [19]. Specifically, deletion of HCN1 in forebrain neurons enhanced short- and long-term memory in mice [19]. Similarly, *Gabra5*−/− mice display better hippocampus-dependent memory performance [13], [23]. Thus, it is possible that reduced I_h_ contributes to the enhanced memory performance of *Gabra5*−/−mice. Additionally, *Gabra5*−/−mice exhibit a reduced sensitivity to memory impairment by etomidate, which potently enhances the activity of α5GABA_A_ receptors [36], [37]. HCN channels are similarly inhibited by anesthetics including propofol and isoflurane [18], and a reduction of I_h_ may also contribute to the reduced sensitivity of *Gabra5*−/− mice to the amnestic effects of anesthetics. Overall, the results of this study suggest a co-regulation of α5GABA_A_ receptors that generate a tonic GABAergic conductance and HCN1 channels that generate I_h_ in hippocampal pyramidal neurons. It will be of future interest to determine whether alterations in I_h_ contribute to the behavioural phenotype of *Gabra5*−/− mice.
